# The Diagnostic and Prognostic Roles Played by Homocysteine and Other Aminothiols in Patients with Chronic Kidney Disease

**DOI:** 10.3390/jcm12175653

**Published:** 2023-08-30

**Authors:** Maria Petrovna Kruglova, Alexander Vladimirovich Ivanov, Anatolij Nikolaevich Fedoseev, Edward Danielevich Virus, Victor Aleksandrovich Stupin, Vladimir Anatolyevich Parfenov, Svetlana Andreevna Titova, Polina Igorevna Lazareva, Aslan Amirkhanovich Kubatiev, Ekaterina Vladimirovna Silina

**Affiliations:** 1 I.M. Sechenov First Moscow State Medical University (Sechenov University), Trubetskaya St., 8, 119991 Moscow, Russia; marykruglova@live.ru (M.P.K.); vladimirparfenov@mail.ru (V.A.P.); honey.liebe@mail.ru (S.A.T.); p.lazareva2305@gmail.com (P.I.L.); 2 Institute of General Pathology and Pathophysiology, Baltiyskaya St., 8, 125315 Moscow, Russia; ivanov_av82@mail.ru (A.V.I.); edwardvirus@yandex.ru (E.D.V.); niiopp@mail.ru (A.A.K.); 3 City Clinical Hospital No. 24 of Moscow Healthcare Department, Piscovaya St., 10, 127015 Moscow, Russia; fedosseev@yandex.ru; 4 Pirogov Russian National Research Medical University, Ostrovityanova St., 1, 117997 Moscow, Russia; stvictor@bk.ru

**Keywords:** chronic kidney disease (CKD), aminothiols, cysteine, homocysteine, s-adenosylmethionine, s-adenosylhomocysteine, capillary electrophoresis

## Abstract

We examined standard clinical and laboratory biochemical parameters, as well as the levels of aminothiols in the blood and urine (homocysteine (Hcy), cysteine (Cys), S-adenosylmethionine (SAM), and S-adenosylhomocysteine (SAH)) via capillary electrophoresis in patients with CKD at stages II–V. Patient outcomes were assessed after five years. To complete forecasting, correlation and ROC analysis were performed. It was found that the levels of Cys and Hcy in blood plasma were earlier markers of CKD starting from stage II, while the levels of SAM and SAM/SAH in urine made it possible to differentiate between CKD at stages II and III. Blood plasma Hcy and urinary SAM and SAM/SAH correlated with mortality, but plasma Hcy concentrations were more significant. Thus, plasma Hcy, urine SAM, and SAM/SAH can be considered to be potential diagnostic and prognostic markers in patients with CKD.

## 1. Introduction

Chronic kidney disease (CKD) is a condition characterized by progressive loss of kidney function over several months or years. The pathogenesis of kidney damage includes a complex set of mechanisms, in which circulatory (arterial hypertension (AH), coronary heart disease (CHD), etc.) and dysmetabolic disorders (diabetes mellitus (DM), gout, etc.) occur, which, in turn, are the most common causes of renal dysfunction [1,2,3,4]. CKD is also associated with the risk of developing both chronic and acute renal failure [5] and cardiovascular complications (heart attacks, strokes, etc.) [6,7]. In this regard, the prevalence of CKD is comparable to the prevalence of these diseases, and it is more than 10% [8], which determines its clinical, social, and economic significance; thus, the early diagnosis and prognosis of the course of the disease are of particular importance. At the moment, in clinical practice, to perform the diagnosis of CKD, the determination of the blood plasma creatinine level is mainly used, followed by the calculation of GFR, which can be normal or increased in the early stages of CKD and is not always able to reflect real changes and the severity of the disease [9,10,11], but remains an important guideline. There are a number of other blood and urine markers of renal dysfunction that appear to be promising in both the diagnosis and prognosis of CKD, such as cystatin C, neutrophil gelatinase-associated lipocalin (NGAL), interleukin-18, kidney injury molecule-1 (KIM-1), monocyte chemoattractant protein-1 (MCP-1), α-1 microglobulin and β-2 microglobulin, urine epidermal growth factor (EGF), and many others [12,13,14,15,16,17]. However, the search for potential diagnostic and prognostic markers of CKD is still relevant.

Thanks to the development of proteomic diagnostic methods, it has become possible to determine protein metabolites in the blood and urine, which not only reflect the dynamics of the development of renal dysfunction, but are also directly involved in the pathogenesis of these disorders, including metabolites of the methionine cycle. Methionine can exogenously enter the body through consumption of food or be formed as a result of the breakdown of endogenous protein. Methionine is then converted into S-adenosylmethionine (SAM) by the enzyme SAM synthase, which is, in turn, converted into S-adenosylhomocysteine (SAH), which is subsequently hydrolyzed by SAH hydrolase to form Hcy and adenosine. Hcy can be utilized in two ways—remethylation (RM) or trans-sulfonation (TS)—followed by regeneration into methionine or conversion into cysteine, respectively [18,19,20]. In recent studies, data from experimental and clinical studies have been presented, indicating the association of homocysteine (Hcy) [21,22,23,24,25,26], as well as its precursors SAM and SAH [27,28,29,30,31], with the development of acute and chronic renal failure. At the same time, the mechanisms of methionine cycle disruption in CKD are not fully understood and may be associated with a decrease in the elimination of these metabolites, as well as the violation of their renal and systemic metabolism [32,33,34]. It is known that, normally, Hcy is almost completely (99%) reabsorbed from primary urine, and in CKD, its clearance is reduced by about 30% [28,29,35], while the clearance of S-adenosylmethionine (SAM) and S-adenosylhomocysteine (SAH) is quite high, normally amounting for 93% and 39%, respectively [29,36]. In connection to the foregoing, these metabolites can be considered to be promising markers that reflect the dynamics of renal dysfunction and dysmetabolic disorders, and they can also be used to determine content in the urine, because non-invasive methods are simple and atraumatic in nature. The aim of our study was to evaluate the diagnostic and prognostic roles played by these markers in blood (Hcy, Cys) and urine (Hcy, Cys, SAM, SAH) at different stages of CKD.

## 2. Materials and Methods

### 2.1. Ethical Approval

All procedures performed in human studies were carried out in accordance with the ethical standards of the institutional and/or national research committee, as well as the Declaration of Helsinki (1964) and its later amendments or comparable ethical standards. This study’s protocol was approved by the Ethics Committee of the Clinical Hospital No. 24 of the Moscow Health Department, where all patients included in this study were hospitalized and treated (protocol No. 6, 16 December 2021).

### 2.2. Patients and Controls

The study was conducted from 18 September 2017 to 30 November 2017. The inclusion criteria for the study were as follows: being aged over 18 years old, a history of chronic kidney disease at stages II–V for more than one year, and obtaining written informed consent to participate in this study from the patient or their relatives. The exclusion criteria of this study were as follows: pregnancy, lactation, HIV infection, viral hepatitis, non-vascular diseases of the central nervous system, severe concomitant somatic pathology (cancer, severe chronic respiratory failure, liver failure, stroke, or myocardial infarction during the previous 4 months), and severe purulent-septic diseases. The termination criteria of the study were as follows: the occurrence of any of the conditions included in the exclusion criteria, violation of the protocol, the request of the patient or their relatives to be withdrawn from this study, and the occurrence of a phenomenon forcing the termination of this study.

### 2.3. Clinical and Instrumental Methods

Upon admission, patients anamnestic, measured pulse, respiratory rate, blood pressure (BP), height, and weight data were collected, followed by the calculation of body mass index (BMI) (weight (kg)/height (m^2^)). In the presence of arterial hypertension (AH), special attention was paid to the gradation of such parameters, such as the level of blood pressure (data on the maximum, adapted, and actual blood pressures were included in the study), as well as the duration and stages of hypertension. Also, to assess the state of the cardiovascular system, an ECG (Electrocardiograph Philips Page Writer TC30, Andover, MA, USA), ECHO-KG (Ultrasound Imaging System EPIQ-7 (Philips Ultrasound, Inc, Bothell, WA, USA) was used. Ultrasound was performed on the abdominal organs using the B-K Medical Pro Focus 2202 ultrasound imaging system (Herlev, Denmark).

In our study, we classified CKD stages according to GFR, which was calculated using the CKD–EPI–creatinine formula (Chronic Kidney Disease Epidemiology Collaboration Formula), and divided them into three groups: 1—patients with CKD at stage II (moderate decrease in GFR); 2—patients with CKD at stages IIIa–IIIb (pronounced decrease in GFR), 3: patients with CKD at stages IV–V (a severe decrease in GFR and pronounced decrease in kidney function). To assess the clinical condition of patients in relation to the underlying disease, standard methods of instrumental and laboratory examination were used, which consisted of identifying markers of damage, structural, and functional changes in the kidneys (including a general urinalysis, daily urinalysis, urinalysis using the Nechoporenko method, and the determination of biochemical blood plasma markers (creatinine, urea, uric acid, total protein, albumin, etc.). A standard clinical and laboratory examination was supplemented by a study of aminothiol levels in urine and blood. The comparison group consisted of healthy people (*n* = 50), who were comparable in terms of sex and age to patients in the CKD group, which was confirmed through a ultrasound study of the kidneys, biochemical blood test, and urine test.

### 2.4. Sample Collection

Venous blood and urine samples were collected from volunteers and controls on the second day after elective hospitalization. Venous blood samples were collected from fasting volunteers in the morning using the cubital vein into a tube with 0.38% sodium citrate and centrifuged for 3 min at 3000× *g*. Then, blood plasma was taken and immediately frozen at −75 °C for further analysis. Urine samples were collected via the Nechiporenko method, and 10 mL was then placed into a test tube with 1 mL of 100-mm hydrochloric acid and centrifuged for 5 min at 10,000× *g*, before being frozen at −80 °C for further analysis.

### 2.5. Laboratory Procedures

Cysteine (Cys), homocysteine (Hcy), S-adenosylmethionine (SAM), and S-adenosylhomocysteine (SAH) were determined using a 3D capillary electrophoresis (CE) system (Agilent Technologies, Waldbronn, Germany), along with a quartz capillary with a total length of 32 cm, an effective length of 23.5 cm, and an inner diameter of 50 µm, as well as with UV detection.

#### 2.5.1. Determination of Cys and Hcy in Plasma and Urine

Aliquots of blood plasma and urine (100 µL) were first derivatized using 50 mM of 1,1-thiocarbonyldiimidazole (10 µL), and 250 µM of penicillamine (10 µL) was added as an internal standard, followed by incubation at 37 °C for 15 min. The samples were then purified via extraction using 50 mM of 1,1-thiocarbonildiimidazole in acetonitrile (300 µL) and incubated for 30 min at 4 °C, followed by centrifugation for 5 min at 15,000× *g*. To perform liquid–liquid extraction, 0.1 mL of chloroform and 20 μL of 1-molarity HCl were added to 0.2 mL of the sample, and the mixture was centrifuged for 1 min at 3000× *g*. The upper phase was discarded, and the lower phase was concentrated in vacuo at 30 °C for 30 min. Then, 50 μL of water was added to the samples and incubated for 5 min, the samples were centrifuged for 1 min at 3000× *g*, and 30 μL of the solution was taken for analysis. Electrophoretic separation was performed using 0.1-molarity phosphate with 30 mM of triethanolamine containing 25 μM of Stabilite, 2.5 of μM sodium dodecyl sulfonate, and 2.5% (*v*/*v*) polyethylene glycol-600 at pH = 2. Samples were introduced into the capillary at 50 mbar for 45 s, and 0.5-molarity KOH at −17 kV was injected over a period of 30 s. The absorption wavelength was 285 nm [37].

#### 2.5.2. Determination of SAM and SAH in Urine

Samples were centrifuged for 1 min at 1000× *g* after thawing. Na_2_HPO_4_ (0.2 mol/L, 0.2 mL) was added to 3.0 mL of urine sample and passed through a solid-phase extraction cartridge grafted using phenylboronic acid (Bond Elut PBA 100 mg phase (Agilent Technologies, Inc., Santa Clara, CA, USA). Next, 10 mg of the Bond Elute-PBA phase (Agilent Technologies, Inc, Santa Clara, CA, USA) was washed with 30% acetonitrile containing 1% (*v*/*v*) formic acid (0.2 mL), water (0.4 mL), and 50 mM of Na-phosphate buffer (pH 8.0; 0.4 mL). A sample (0.4 mL) was loaded, and the phase was washed with 50 mM of Na-phosphate buffer (pH 8.0; 0.4 mL) and 10 mM of Na-phosphate buffer (pH 8.0; 0.4 mL). The analytes were desorbed using HCl (0.1 M, 0.2 mL). Before the start of the experiment, the capillary was washed daily with 1-molarity sodium hydroxide (2 min), water (1 min), and a supporting electrolyte solution (0.05-molarity sodium dodecyl sulfate, 0.05-molarity H_3_PO_4_, 5% (*v*/*v*) isobutanol, 10% (*v*/*v*) polyethylene glycol-300) (3 min). A urine sample was injected for 120 s at 50 mbar, followed by a sample of micellar solution (215 mM of sodium dodecyl sulfate, 50 mM of H_3_PO_4_, and 10% (*v*/*v*) polyethylene glycol-300), which was injected for 45 s at 50 mbar at −11 kV for the first 2.5 min, followed by −16 kV for the remaining 4.5 min. The capillary was then flushed with BGE for 0.5 min. Analysis was performed at a pH of 2.2. The absorption was 254 nm [38,39]. The SAM/SAH ratio was calculated by dividing the SAM concentrations by the SAH concentrations.

### 2.6. Outcome

To make a prediction, we collected follow-up data 5 years after hospitalization by interviewing patients and/or their relatives (all patients), as well as examining and analyzing the data of 61% of patients. The catamnesis data were collected from 19 September 2022 to 31 October 2022.

### 2.7. Statistical Analysis

Primary processing of electropherograms was carried out using Data Analysis ChemStation software, version B.01.03 (Agilent Technologies).

When planning the study, the following setting characteristics were used to calculate the minimum sample size: a type I error of no more than 5% (reliability a = 0.05) and power of 80% (β = 1 − 0.8 = 0.2). Data were collected and analyzed using the statistical program Statistical Package for the Social Sciences SPSS 23.0 software, (IBM Company, Armonk, NY, USA). The normality of data distribution was preliminarily checked (Kolmogorov–Smirnov test) and, since not all data were normally distributed, it was expressed as a median (between the 1st and 3rd quartiles). Correlations were performed using Spearman’s (ρ) rank testing. Differences between groups were tested for significance using the non-parametric Mann–Whitney U test for comparison of two groups and the Kruskal–Wallis test (taking into account the Bonferroni correction for multiple comparisons) for three or more groups. Qualitative indicators (nominal and ordinal) were analyzed according to the contingency table and the Chi-squared test. The receiver operating characteristic (ROC) curve was used to estimate predictors of CKD at various stages, as well as to assess the predictive value of the CKD outcome. Also, to determine the prognostic significance of the indicators that we studied, we used the Cox regression method (the backward Wald method). The observed difference was considered to be significant at *p* < 0.05.

## 3. Results

### 3.1. Patient Characteristics

The study included 110 patients with chronic kidney disease at stages II–V, and 50 patients were included in the control group. The mean age of the main study group was 67 years old (56–76 years old); 41.8% of these patients were men. Moreover, 33 patients had stage II CKD, 46 patients had CKD at stages IIIa–IIIb, and 31 patients had CKD at stages IV–V. The mean GFR in the study group was 44.1 (28.0; 64.8) mL/min/1.73 m^2^, and the mean plasma creatinine concentration was 118.0 (96.0; 198.0) µmol/L. The average duration of the underlying disease was two years (1; 5). Edema was observed in 40.9% of patients. Also, increases in the concentrations of markers of kidney damage, such as urea and uric acid, and decreases in the level of blood plasma albumin and hemoglobin were observed in the blood, while the amount of daily protein present in the urine increased (Table 1).

### 3.2. Changes in Aminothiol Levels Depending on the Histories of CKD Patients and the Stage of the Disease

When comparing healthy volunteers and patients with CKD, the studied values of Hcy, Cys in blood and SAM, SAM/SAH, Cys, and Hcy in urine were significantly statistically different from each other (Table 2). The average concentrations of SAM, SAM/SAH, Cys, and Hcy in urine were 2.9 (*p* < 0.001), 2.5 (*p* < 0.001), 2.3 (*p* < 0.001), and 1.8 (*p* < 0.001) times lower, respectively, than in the control group, while the concentrations of Cys and Hcy of blood plasma were 1.6 (*p* < 0.001) and 1.9 (*p* < 0.001) times higher, respectively. In contrast, SAH did not show statistically significant differences between these groups.

When comparing the concentrations of aminothiols, depending on the stage of CKD, the severity of changes in their concentrations progressed in proportion to the increase in renal dysfunction (Table 3).

Urine Cr levels significantly differed from those of the control, starting from stage III CKD; on average, its level was 1.7 (*p* < 0.001) times lower in patients with stages IIIa–IIIb of CKD and 2.4 (*p* < 0.001) times lower in patients with IV–V stages of CKD compared to the control group. No other intergroup differences were found. The urine SAM level was lower than that of the control by an average of 2.9 (*p* < 0.001) times in patients with stages IIIa–IIIb of CKD and 4.5 (*p* < 0.001) times in patients at stages IV–V of CKD. Statistically significant differences were also found between stages II and IIIa–IIIb of CKD and stages II and IV–V of CKD: urinary SAM concentrations in patients at stages IIIa–IIIb and stages IV–V of CKD (*p* < 0.001) were 2.1 (*p* = 0.019) and 3.2 (*p* < 0.001) times lower, respectively, than in patients at stage II of CKD. The level of urine ratio of SAM/SAH also significantly differed from the control starting from stage III of CKD. Urine SAM/SAH was lower, on average, by 2.9 times (*p* < 0.001) and 4.3 times (*p* < 0.001) in patients at stages IIIa–IIIb and IV–V of CKD, respectively. Statistically significant differences were also found between stages II and IIIa–IIIb and II and IV–V: the urinary SAH/SAM ratio at stages IIIa–IIIb was 2.4 (*p* = 0.002) times lower and 3.6 (*p* < 0.001) times lower than in stage II patients. Urinary Cys and Hcy levels showed differences to those of controls starting from stage III CKD. Urine Cys concentrations were 3.2 times (*p* < 0.001) lower than those in the control at stages IIIa–IIIb and 2.6 times (*p* < 0.001) lower at stages IV–V, i.e., urine Cys concentrations slightly increased at stages IV–V and showed differences between the second and third stages of CKD (urine Cys of the third stage was 2.7 times lower (*p* < 0.001) than that of the second stage). Urine Hcy was 2.2 times (*p* < 0.001) lower than that of the control at stages IIIa–IIIb and 1.9 times (*p* < 0.001) lower at stages IV–V, though they did not reflect other intergroup differences. The Cys and Hcy of blood plasma were the most sensitive, being significantly different to the control of patients at stage II of CKD, while the level of plasma creatinine significantly differed from the control starting from stage III of CKD (Table 4). Thus, the level of Cys was 1.6 (*p* < 0.001) times higher than in the control group at stages II, IIIa–IIIb, and IV–V. Hcy concentrations were 1.5 (*p* < 0.001), 1.8 (*p* < 0.001), and 2.6 (*p* < 0.001) times higher than in the control group at the second, third, and fourth–fifth stages of CKD, respectively, with At the same time. Hcy concentrations significantly differed between both the II and IV–V stages (*p* = 0.004) and IIIa–IIIb and IV–V stages (*p* = 0.031).

According to the ROC analysis (Table 4), when assessing AUC, the markers were Hcy, blood plasma, µmol/L (AUC = 0.905, *p* = 0.025); Cys, blood plasma, µmol/L (AUC = 0.880, *p* = 0.026); SAM/SAH, urine (AUC = 0.263, *p* = 0.045); Cys, urine, µmol/L (AUC= 0.255, *p* = 0.039); and SAM, urine, µmol/L (AUC = 0.240, *p* = 0.044). It is also worth noting that when using the framework of the non-invasive determination of the studied metabolites in urine, SAM had the greatest potential, while urinary creatinine reflected this potential to a lesser extent (AUC = 0.275, *p* = 0.045). Urine aminothiols showed the greatest diagnostic significance between stages II and IIIa–IIIb of CKD, i.e., transition from a moderate decrease in GFR to a more pronounced decrease: SAM, urine (AUC = 0.282, *p* = 0.001); Cys, urine (AUC = 0.221, *p* < 0.001); and SAM/SAH, urine (AUC = 0.209, *p* < 0.001). The Hcy (AUC = 0.848, *p* < 0.001) and Cys (AUC = 0.857, *p* < 0.000) parameters of blood plasma most optimally demonstrated their diagnostic significance at stage II of CKD, and Hcy reflected the differences between stages IIIa–IIIb and IV–V (AUC =0.756, *p* = 0.057).

### 3.3. Analysis of the Relationship between Aminothiols in Blood Plasma and Urine and the Survival of Patients with CKD after Five Years

As a result of the collection of follow-up data five years after hospitalization, it was found that 31 patients out of 110 died. Indeed, 24 patients died from acute renal failure that developed against the background of CKD, 1 patient died from first-time left ventricular myocardial infarction, 4 patients died from repeat LV infarction, and 2 patients died from stroke. Moreover, 1 patient was initially diagnosed with stage II CKD, 9 patients were diagnosed with stage III CKD, and 20 patients were diagnosed with stage IV or V CKD. In order to assess the predictive ability of aminothiol correlation, ROC analyses were performed with the inclusion of 76 variables (established in all 110 patients), which had relationships with the outcome of CKD. For the selected variables, the area under the curve (AUC) scores were calculated. Regarding the set of variables discussed above, those with good predictive quality (AUC above 0.7 or below 0.3) were selected (Table 5).

Thus, the following 23 indicators reflected a high level of predictive potential: blood urea, mmol/L (AUC = 0.845, *p* < 0.001), plasma creatinine, µmol/L (AUC = 0.836, *p* < 0.001); age, full years (AUC = 0.828, *p* < 0.001); CKD stage (AUC = 0.827, *p* < 0.001); urinary protein, g/L (AUC = 0.772, *p* < 0.001); urine protein/creatinine ratio (PCR), mg/mmol (AUC = 0.760, *p* < 0.001); ESR, mm/h (AUC = 0.754, *p* < 0.001); daily urine protein, g/L (AUC = 0.744, *p* < 0.001); plasma homocysteine, µmol/L (AUC = 0.733, *p* < 0.001); granulocytes, % (AUC = 0.724, *p* < 0.001); adapted systolic blood pressure, mmHg (AUC = 0.717, *p* < 0.001); leukocytes *10^9^/L (AUC = 0.705, *p* = 0.001); segmented neutrophils, % (AUC = 0.703, *p* = 0.001); SAM urine, µmol/L (AUC = 0.292, *p* = 0.026); total bilirubin, µmol/L (AUC = 0.278, *p* < 0.001); SAM/SAH urine (AUC = 0.276, *p* < 0.001); lymphocytes, % (AUC = 0.275, *p* = 0.025); iron, µmol/L (AUC = 0.229, *p* < 0.001); hematocrit, % (AUC = 0.226, *p* < 0.001); daily diuresis, L/day (AUC = 0.217, *p* < 0.001); hemoglobin, g/L (AUC = 0.189, *p* < 0.001); erythrocytes *10^12^/L (AUC = 0.172, *p* < 0.001); and the glomerular filtration rate, mL/min/1.73 m^2^ (AUC = 0.130, *p* < 0.001). The average quality of the prognostic model is set for indicators that have AUCs of 0.6–0.7 or 0.3–0.4. These indicators included plasma cysteine, µmol/L (AUC = 0.608, *p* = 0.045), and urine creatinine, µmol/L (AUC = 0.410, *p* = 0.141).

**Figure 1:**
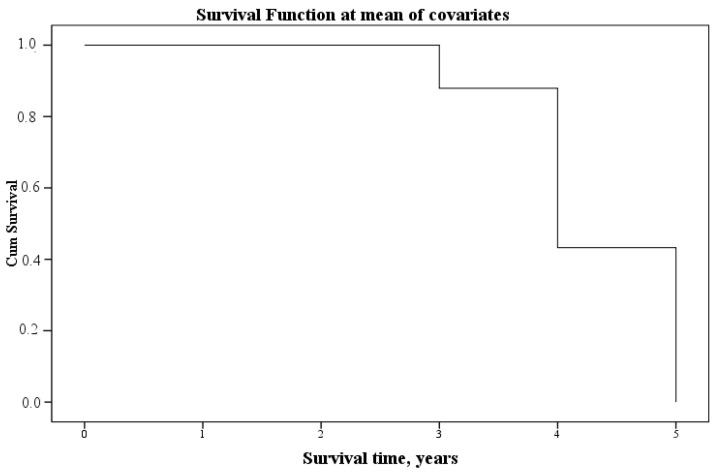
Survival timeline.

We also performed Cox regression to perform survival analysis for two, three, four, and five years. Of the indicators of aminothiols studied in blood plasma and urine, as well as creatinine and urea in blood and urine, at the 10th step of iterations, statistical significance was determined for only one indicator–blood plasma homocysteine. The final calculation formula is presented in Table 6, and the survival graph is presented in Figure 1.

## 4. Discussion

As shown in a number of studies, the kidneys play a leading role in the regulation of the methionine cycle and have a direct effect on changes in the concentrations of Hcy, Cys, SAM, SAH, and the SAH/SAM ratio [40,41,42]. Although the mechanisms driving these disorders in CKD are not fully understood, the strong relationship between Hcy, Cys, SAM, SAH, and plasma creatinine levels and the glomerular filtration rate (GFR) [30,32,43,44] initially suggested that these metabolites were excreted in the urine through glomerular filtration. However, there are data indicating the presence of enzymes of the trans-sulfonation (TS) and remethylation (RM) pathways in human renal tissue, which indicates the possible involvement of a renal metabolism [33]. According to the data obtained by Correia et al. [45], the kidneys are the most cysteine-rich organ; therefore, its increase in the blood in case of kidney damage may also be an earlier marker than the change in GFR. Thus, according to the results of our study, the levels of Cys and Hcy in blood plasma already began to increase at stage II CKD and made it possible to reliably distinguish between group of healthy volunteers and patients with stage II CKD, as well as plasma and urine creatinine, SAM, SAM/SAH, Cys, and Hcy urine from stage III CKD, which is consistent with our previous studies [28,29]. Changes in the concentrations of Cys and Hcy in blood plasma were earlier signs, suggesting that, initially, there is still a primary disturbance in the metabolism of aminothiols until a more pronounced decrease in GFR and a violation of the elimination of these metabolites occur, while, based on the fact that the level of Cys in also increased, trans-sulfonation reactions in these patients were not disturbed, which is also consistent with a number of prior studies [46,47,48].

It is known that disturbances in the methionine cycle are associated not only with kidney damage [49], but also with a number of other conditions, such as inflammation [50,51], atherosclerosis [52], endothelial dysfunction [53], dysregulation of DNA methylation and gene expression [54], etc., which greatly increase the risk of complications and death. When evaluating the five-year survival rate in patients based on the markers we studied, SAM and SAM/SAH of urine showed a predictive potential, but plasma homocysteine had the highest prognostic potential, which is comparable to the data of other studies. [55,56].

## 5. Conclusions

Plasma Cys and Hcy levels were earlier markers of CKD and made it possible to identify kidney damage from stage II CKD onward, demonstrating higher sensitivity than plasma creatinine. In contrast, when performing non-invasive diagnostics in the urine, the levels of SAM and SAM/SAH made it possible to distinguish stage II CKD from stage III CKD. When assessing survival, these markers showed a good prognostic result; however, high plasma homocysteine concentrations were more significant.

## Figures and Tables

**Table 1:** Major characteristics of kidney function in patients with CKD (according to the stages of CKD).

Variables	All	Stage II	Stages IIIa–IIIb	Stages IV–V	*p*-Value
General characteristics					
N	*n* = 110	*n* = 33 (30%)	*n* = 46 (41.8%)	*n* = 31 (28.2%)	-
Gender, men	*n* = 46 (41.8%)	*n* = 17 (51.5%)	*n* = 17 (36.9%)	*n* = 12 (38.7%)	0.594
Age, full years	67 (56; 76)	52 (38.5; 61.5)	70.5 (59; 78) *	74 (67; 79) *	<0.001
BMI, kg/m^2^	28.9 (23.4; 34.0)	26.2 (23.0; 32.8)	29.4 (24.8; 34.3)	29.4 (22.1; 33.1)	0.335
Obesity	*n* = 45 (40.9%)	*n* = 11 (33.3%)	*n* = 20 (43.5%)	*n* = 14 (45.2%)	0.565
Edema	*n* = 32 (29%)	*n* = 6 (18%)	*n* = 12 (26%)	*n* = 14 (45%)	<0.001
Duration of CKD, years	2 (1; 5)	1 (1; 3.5)	2 (1; 4)	1 (1; 5)	0.609
Clinical and laboratory parameters					
Blood plasma creatinine, µmol/L	118.0 (96.0; 198.0)	85.0 (83.0; 97.0)	118.5 (107.0; 144.0) *	357.0 (245.5; 481.0) *	<0.001 IIIa–IIIb–IV–V (0.001)
GFR, mL/min/1.73 m^2^	44.1 (28.0; 64.8)	74.4 (68.2; 80.7)	43.5 (38.0; 49.2) *	10.6 (8.35; 19.6) *	<0.001 IIIa–IIIb–IV–V (0.001)
Uric acid, µmol/L	399.5 (314.0; 483.0)	315.0 (247.0; 403.0)	403.0 (340.0; 465.0) *	462.0 (374.5; 564.0) *	<0.001
Urea, µmol/L	8.1 (5.2; 14.6)	4.6 (3.8; 5.4)	8.3 (6.5; 10.7)	18.0 (15.7; 23.4) *	<0.001 IIIa–IIIb–IV–V (<0.001)
Sodium, mmol/L	142.0 (141.0; 144.0)	142.0 (141.0; 144.0)	143.5 (142.0; 145.0)	141.0 (138.5; 143.5) *	<0.001 IIIa–IIIb–IV–V (<0.001)
Potassium, mmol/L	4.6 (4.3; 5.0)	4.4 (4.2; 4.6)	4.6 (4.3; 4.9)	5.0 (4.5;5.4) *	<0.001
Blood plasma albumin, g/L	42.4 (38.9; 45.8)	43.6 (40.9; 47.2)	42.8 (40.8; 45.7)	38.0 (35.2; 42.3) *	<0.001 IIIa–IIIb–IV–V (0.003)
Daily urine protein, g/L	0.2 (0.1; 0.8)	0.1 (0.1; 0.2)	0.1 (0.1; 0.4)	0.9 (0.3; 2.7) *	<0.001 IIIa–IIIb–IV–V (<0.001)
Hemoglobin g/L	127.5 (107.0; 144.0)	144.0 (134.0; 147.0)	134.0 (117.0; 145.0)	101.0 (90.0; 116.5) *	<0.001

For qualitative indicators (nominal and ordinal), the *p*-value of a Chi-squared test is presented. The medians and the interquartile interval with Tukey’s folds are shown. *—difference from stage II CKD at *p* < 0.05 (Kruskal–Wallis test), taking into account Bonferroni’s correction for multiple comparisons.

**Table 2:** Changes in the parameters of aminothiols and creatinine levels in urine and blood plasma between control group and patients with CKD.

Variables	Controls (*n* = 50)	CKD (*n* = 110)	*p*-Value (Mann–Whitney Test)
urine			
Cr, µmol/L	3.9 (2.5; 5.4)	2.3 (1.3; 3.3)	<0.001 *
SAM, µmol/L	9.5 (6.1; 12.1)	3.3 (1.7; 5.9)	<0.001 *
SAH, µmol/L	0.9 (0.4; 1.6)	1.1 (0.5; 1.7)	0.262
SAM/SAH	8.2 (3.7; 26.0)	3.3 (1.9; 6.8)	<0.001 *
Cys, µmol/L	136.8 (95.7; 211.1)	59.0 (31.6; 120.9)	<0.001 *
Hcy, µmol/L	2.9 (1.8; 4.5)	1.6 (0.8; 2.9)	<0.001 *
blood			
Cr, µmol/L	76.0 (71.0; 88.0)	118.0 (96.0; 198.0)	<0.001 *
Cys, µmol/L	264.0 (214.4; 303.2)	420.1 (331.4; 513.1)	<0.001 *
Hcy, µmol/L	10.9 (8.6; 12.5)	20.9 (15.9; 28.8)	<0.001 *

The medians and the interquartile interval with Tukey’s folds are shown. *—difference between the control and CKD groups at *p* < 0.05.

**Table 3:** Changes in the parameters of aminothiols and creatinine levels in urine and blood plasma, depending on the stage of CKD.

Variables	Control Group (*n* = 50)	CKD at Stage II (*n* = 33)	CKD at Stages IIIa–IIIb (*n* = 46)	CKD at Stages IV–V (*n* = 31)	*p*-Value (Kruskal–Wallis Test)
urine					
Cr, µmol/L	3.9 (2.5; 5.4)	2.8 (1.4; 3.5)	2.3 * (1.5; 3.4)	1.6 * (1.3; 2.7)	<0.001
SAM, µmol/L	9.5 (6.1; 12.1)	6.7 (4.3; 9.2)	3.2 * (1.8; 5.5)	2.1 * (1.5; 2.8)	<0.001 II–IIIa–IIIb (0.019); II–IV–V (0.000)
SAH, µmol/L	0.9 (0.4; 1.6)	1.0 (0.6; 1.5)	1.3 (0.5; 1.7)	0.9 (0.6; 1.9)	0.694
SAM/SAH	8.2 (3.7; 26.0)	6.8 (4.7; 8.6)	2.8 * (1.9; 4.8)	1.9 * (1.2; 2.8)	<0.001 II–IIIa–IIIb (0.002); II–IV–V (0.000)
Cys, µmol/L	136.8 (95.7; 211.1)	117.6 (54.8; 190.8)	42.8 * (26.0; 70.4)	53.6 * (29.9; 128.3)	<0.001 II–IIIa–IIIb (0.000)
Hcy, µmol/L	2.9 (1.8; 4.5)	2.5 (1.3; 3.3)	1.3 * (0.7; 2.5)	1.5 * (1.0; 2.5)	<0.001
blood					
Cr, µmol/L	76.0 (71.0; 88.0)	85.0 (83.0; 97.0)	118.5 * (107.0; 144.0)	357.0 * (245.5; 481.0)	<0.001 II–IIIa–IIIb (0.000); II–IV–V (0.000); IIIa–IIIb–IV–V (0.001)
Cys, µmol/L	264.0 (214.4; 303.2)	410.3 * (319.4; 513.1)	424.2 * (334.7; 471.5)	413.7 * (336.4; 569.8)	<0.001
Hcy, µmol/L	10.9 (8.6; 12.5)	16.7 * (13.5; 22.7)	19.2 * (15.7; 23.3)	28.7 * (20.8; 47.9)	<0.001 II–IV–V (0.004); IIIa–IIIb–IV–V (0.031)

The medians and the interquartile interval with Tukey’s folds are shown. *—difference from the control group at *p* < 0.05, taking into account Bonferroni’s correction for multiple comparisons.

**Table 4:** Results of the ROC analysis of predictive potential of aminothiols at various stages of CKD.

Control–CKD	AUC	*p*	Control–Stage II of CKD	AUC	*p*
Cr, blood plasma, µmol/L	0.915	0.022	Cys, blood plasma, µmol/L	0.857	0.000
Hcy, blood plasma, µmol/L	0.905	0.025	Hcy, blood plasma, µmol/L	0.848	0.000
Cys, blood plasma, µmol/L	0.880	0.026	Cr, blood plasma, µmol/L	0.755	0.000
SAH, urine, µmol/L	0.555	0.050	SAH, urine µmol/L	0.538	0.564
Hcy, urine, µmol/L	0.310	0.043	SAM/SAH, urine, µmol/L	0.416	0.199
Cr, urine, µmol/L	0.275	0.045	Hcy, urine, µmol/L	0.409	0.161
SAM/SAH, urine	0.263	0.045	Cys, urine, µmol/L	0.405	0.147
Cys, urine, µmol/L	0.255	0.039	SAM, urine, µmol/L	0.388	0.086
SAM, urine, µmol/L	0.240	0.044	Cr, urine, µmol/L	0.323	0.007
CKD at stages II and IIIa–IIIв	AUC	*p*	CKD at stages IIIa–IIIb and IV–V	AUC	*p*
Cr, blood plasma, µmol/L	0.938	0.000	Cr, blood plasma, µmol/L	0.985	0.011
Hcy, blood plasma, µmol/L	0.567	0.311	Hcy, blood plasma µmol/L	0.756	0.057
SAH, urine, µmol/L	0.531	0.644	Cys, urine, µmol/L	0.572	0.068
Cys, blood plasma, µmol/L	0.495	0.937	Hcy, urine, µmol/L	0.559	0.067
Cr, urine, µmol/L	0.440	0.366	Cys, blood plasma, µmol/L	0.531	0.070
Hcy, urine, µmol/L	0.336	0.013	SAH, urine, µmol/L	0.509	0.067
SAM, urine, µmol/L	0.282	0.001	Cr, urine, µmol/L	0.392	0.066
Cys, urine, µmol/L	0.221	0.000	SAM/SAH, urine, µmol/L	0.327	0.064
SAM/SAH, urine, µmol/L	0.209	0.000	SAM, urine, µmol/L	0.325	0.061

**Table 5:** Area under the curve (AUC) and correlation analysis of prognostic variables.

№	Variables	Survival (Spearman Correlation Coefficient)	AUC	*p*
1	Blood plasma urea, mmol/L	0.538 **	0.845	0.000
2	Blood plasma creatinine, µmol/L	0.746 **	0.836	0.000
3	Age, full years	0.588 **	0.828	0.000
4	CKD stage	0.577 **	0.827	0.000
5	Protein in urine, g/L	0.425 **	0.772	0.000
6	Urine protein/creatinine ratio (PCR), mg/mmol	0.405 **	0.760	0.000
7	ESR, mm/h	0.396 **	0.754	0.000
8	Daily urine protein, g/L	0.380 **	0.744	0.000
9	Blood plasma homocysteine, µmol/L	0.685 **	0.733	0.000
10	Granulocytes, %	0.349 **	0.724	0.000
11	Arterial pressure systolic adapted, mm Hg.	0.351 **	0.717	0.000
12	Leukocytes * 10^9^/L	0.320 **	0.705	0.001
13	Segmented neutrophils, %	0.317 **	0.703	0.001
14	SAM urine, µmol/L	−0.314 **	0.292	0.006
15	Bilirubin total, µmol/L	−0.347 **	0.278	0.000
16	SAM/SAH urine	−0.356 **	0.276	0.001
17	Lymphocytes, %	−0.350 **	0.275	0.025
18	Iron, µmol/L	−0.422 **	0.229	0.000
19	Hematocrit, %	−0.427 **	0.226	0.000
20	Daily diuresis, L/day	−0.441 **	0.217	0.000
21	Hemoglobin, g/L	−0.485 **	0.189	0.000
22	Red blood cells * 10^12^/L	−0.511 **	0.172	0.000
23	Glomerular filtration rate, mL/min/1.73 m^2^	−0.576 **	0.130	0.000

** Correlation is significant at the 0.01 level (two tailed). * Correlation is significant at the 0.05 level (two tailed).

**Table 6:** Variables in the Equation.

	B	SE	Wald	df	Sig.	Exp(B)	95% CI for Exp(B)	
							Lower	Upper
Blood plasma homocysteine, µmol/L	0.029	0.012	5.353	1	0.021	1.029	1.004	1.054

## Data Availability

Data will be made available upon reasonable request by the corresponding author. Data are not publicly available due to privacy or ethical restrictions.
